# Scalable loading of a two-dimensional trapped-ion array

**DOI:** 10.1038/ncomms13005

**Published:** 2016-09-28

**Authors:** Colin D. Bruzewicz, Robert McConnell, John Chiaverini, Jeremy M. Sage

**Affiliations:** 1 Lincoln Laboratory, Massachusetts Institute of Technology, 244 Wood Street, Lexington, Massachusetts 02420, USA

## Abstract

Two-dimensional arrays of trapped-ion qubits are attractive platforms for scalable quantum information processing. Sufficiently rapid reloading capable of sustaining a large array, however, remains a significant challenge. Here with the use of a continuous flux of pre-cooled neutral atoms from a remotely located source, we achieve fast loading of a single ion per site while maintaining long trap lifetimes and without disturbing the coherence of an ion quantum bit in an adjacent site. This demonstration satisfies all major criteria necessary for loading and reloading extensive two-dimensional arrays, as will be required for large-scale quantum information processing. Moreover, the already high loading rate can be increased by loading ions in parallel with only a concomitant increase in photo-ionization laser power and no need for additional atomic flux.

## Abstract (web summary)

Two-dimensional arrays of trapped ion qubits are attractive platforms for quantum information processing, but rapid reloading remains a challenge. Here the authors use a continuous flux of pre-cooled neutral atoms to achieve fast loading of single ions without affecting the coherence of adjacent qubits.

## Graphical abstract

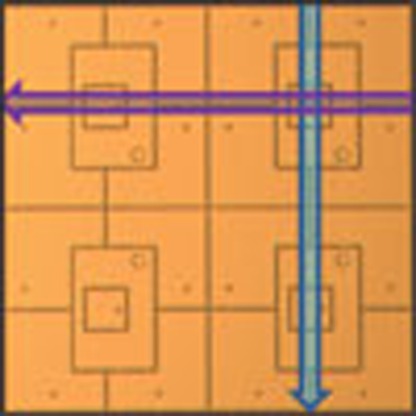

Trapped ions have the potential to form the basis of a large-scale quantum processor due to ion internal states' natural isolation from environmental disturbances and to the straightforward, high-fidelity methods developed to manipulate those states^1^. However, arrays of many ions will require site reloading when an ion is lost due to collisions or reactions with background gas species. Even in cryogenic vacuum systems with single-ion lifetimes greater than tens of hours^2^, arrays of ions approaching the physical qubit count required for practical fault tolerant operation^3^,^4^ will potentially require continuous reloading of empty sites at an average rate much greater than 1 s^−1^. In addition, ions will be lost at random locations throughout the array, necessitating random-access loading at high rates. The ion reloading process must also not lead to unacceptable levels of decoherence in nearby trapped-ion qubits. Otherwise, fault tolerance may be compromised.

Refilling an array from loading zones at the array's edge is limited by the time required to move an ion to interior sites and requires additional complexity in the trap electrode structure to transport ions throughout the array. If ions are instead introduced into the array only at the edge to eliminate these requirements^5^, a large overhead of quantum-logical-swap operations that scales poorly with array size is accrued. Loading ions through holes in the chip^6^ may potentially be implemented with many holes near the array sites to allow rapid random access, but this would likely preclude the on-chip integration of electronic and photonic components necessary for scalable control and readout across an array.

Here, we demonstrate two-dimensional (2D) ion-trap array loading that, uniquely among implemented or proposed methods, satisfies the key requirements for scalability to large numbers of ions. Using a continuous, pre-cooled, neutral atomic beam^7^, which is amenable to loading shallow surface-electrode traps with high isotopic selectivity^8^, we rapidly load sites with random access and without moving any ions in or through the array. The spatial separation of the pre-cooled atom source from the ion-trap array allows for a continuous cold-atom flux, while still providing long ion lifetimes in scalable planar traps. We also show that site-specific ion loading can be accomplished while introducing in neighbouring sites only a small qubit memory error. This error, which arises from photo-ionization beam pointing fluctuations, can be eliminated via standard laser stabilization techniques^9^. Therefore, quantum processing in other parts of the array may continue during ion replacement without additional error, allowing for fault-tolerant operation. The method demonstrated here is an enabling capability for practical operation of large-scale trapped-ion quantum information processors.

## Results

### Ion-trapping system

**Figure 1:**
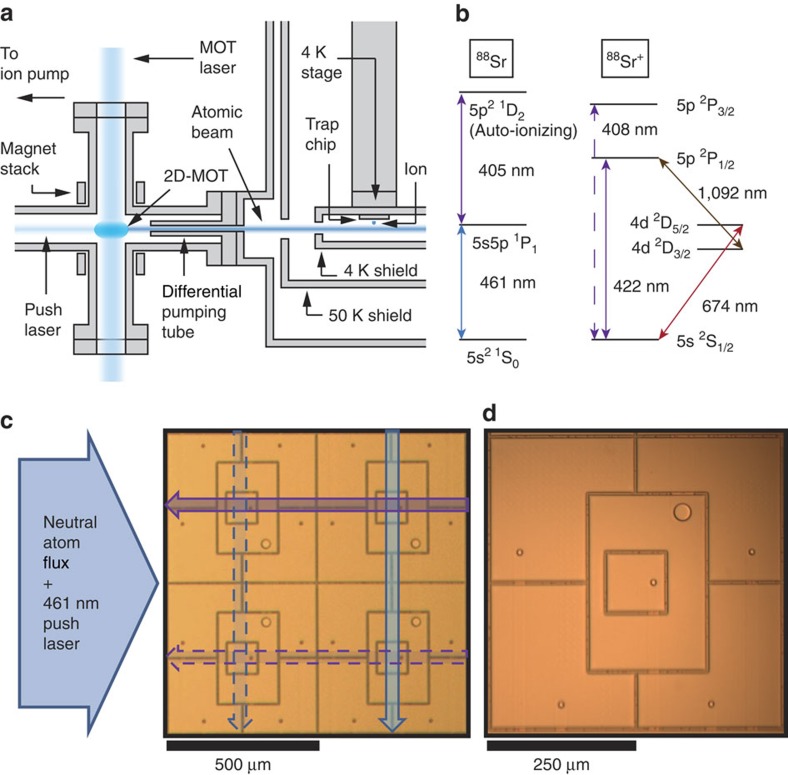
Strontium ion loading and trapping. (**a**) Simplified schematic of 2D-MOT. A cooled atomic beam of neutral strontium propagates from the 2D-MOT to the cryogenic ion-trapping chamber. (**b**) Relevant transitions for the laser cooling and manipulation of neutral and singly-ionized ^88^Sr. Energy splittings not drawn to scale. (**c**) Schematic representation of the site-selective loading scheme overlaid on a micrograph of the 2 × 2 trap array. 461 nm (vertical/blue) and 405 nm (horizontal/violet) PI beams propagate orthogonally to each other such that only the site to be loaded (upper right in this case) is illuminated with both wavelengths. Dashed arrows denote paths of PI beams used to load other array sites. (**d**) Micrograph of an individual trap array site. The rectangular RF ring electrode sufficiently tilts the trap axes to permit efficient Doppler-cooling with a single laser beam. Electrical connections to the trap electrodes are made using the circular interlayer vias.

The cryogenic ion-trapping apparatus used in this work is shown in Fig. 1a and is a variation of a system used previously^8^,^10^,^11^. To achieve fast ion-loading rates and long trap lifetimes, we have implemented a two-dimensional magneto-optical trap (2D-MOT) of neutral strontium operating on the 5*S*_0_→5*P*_1_ transition at 461 nm. Separating the MOT from the cryogenic ion-trapping system permits continuous operation of the atomic oven without limiting ion lifetimes by background gas collisions. Additional details on the 2D-MOT are given in the ‘Methods' section.

Near the ion trap chip, two focused lasers propagating parallel to the trap surface produce ^88^Sr^+^ ions from the cold atomic beam by means of a two-step photo-ionization (PI) process, shown schematically in Fig. 1. A resultant ion is subsequently Doppler cooled and trapped 50 μm from the surface-electrode point-Paul-trap array.

Three-dimensional confinement is provided by a time-varying radio frequency (RF) voltage applied to the rectangular ring electrodes (see Fig. 1d), yielding radial trapping frequencies of a few megahertz^12^. The RF amplitude is adjusted such that only a single ion can be trapped in each site. Operating in this stability regime reduces the average trap lifetime to a few hours, but lifetimes >18 h in the presence of Doppler-cooling light have been observed at lower RF amplitude. Segmented DC electrodes inside and surrounding the RF electrode are used to adjust the location of the ion and compensate for stray electric fields.

The trap array consists of four separate traps arranged in a 2 × 2 square geometry with an array pitch of 500 μm. Although ion-ion Coulomb interactions are too small at this distance for practical multi-qubit logic gates, we expect future designs will be of a similar size with additional electrodes to permit shuttling of ions to adjacent sites to increase the interaction strength when performing two-qubit gates^13^. The additional space afforded by this array pitch may also permit the use of integrated photonic devices to route the large number of laser beams needed for scalable operation. The trap chip fabrication process is described further in the ‘Methods' section.

### Site-selective loading

To avoid additional computational overhead, scalable loading of a 2D ion-trap array must be site-selective. We achieve this by aligning the 461 and 405 nm lasers that drive the two-step PI orthogonally to each other such that only the chosen site to be loaded is addressed by both necessary wavelengths. We are able to quickly switch the locations of the PI lasers to address any desired site by changing the driving frequencies and deflection angles of steering acousto-optic modulators (AOM) in the laser beam paths^14^. In our current geometry, AOM shifts of 50–60 MHz are sufficient to achieve the necessary 500 μm beam translations between array sites. The 461 nm laser is sent through an additional AOM in a double-pass configuration that keeps the laser frequency on resonance as the steering AOM frequency is changed. The auto-ionizing transition at 405 nm is sufficiently broad that such frequency compensation is not necessary for this beam.

We determined the ion-loading rates by measuring the loading probability of a chosen array site as a function of PI time. To achieve the highest loading rates, the 2D-MOT and push laser beams ran continuously such that neutral atoms were always available to be photo-ionized at the trap locations. In each trial, the PI laser beams were pulsed on for a variable time, followed by a 2 ms pulse of 422 nm Doppler-cooling light. We then waited 8 ms without Doppler-cooling to ensure that any transiently trapped ions had left the trap. Following this delay, we measured resonant fluorescence during a second 2 ms Doppler-cooling pulse to detect the presence of a single, stably trapped ion. After detection, a modest positive voltage (1 V) was applied for 1 ms to the centre square DC electrode to eject any trapped ion. An ion-repumping laser at 1,092 nm (see Fig. 1b) also illuminated the array throughout the loading experiments.

**Figure 2:**
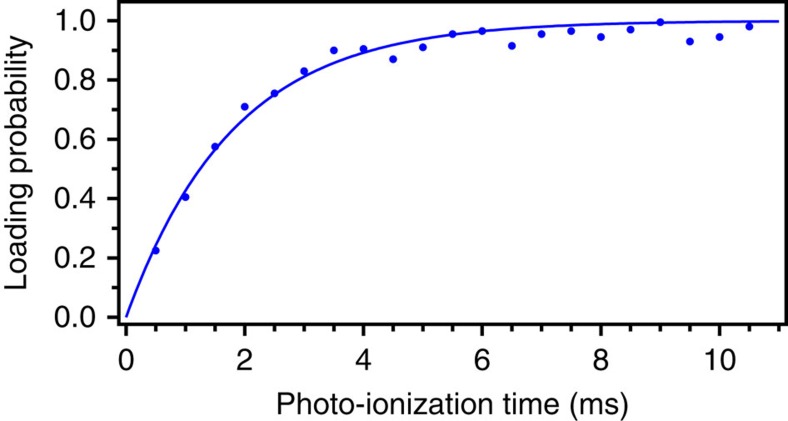
Loading rate of a single ion-trap array site. The upper-right site in Fig. 1c was illuminated with both the 461 and 405 nm lasers for the variable photo-ionization time, and the presence or absence of an ion in the trap was subsequently determined. Each trial was repeated 200 times per point to find the loading probability. Fit to model 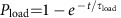 yields a time constant 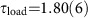
ms.

These trials were repeated 200 times for each PI load time to determine the loading probability. The trap loading rate was characterized by fitting the loading probability as a function of PI time to an exponential model 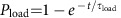. From this fit, we define an average loading rate 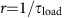 for the Poissonian loading process. The results of these measurements are given in Fig. 2 and Table 1, showing average loading rates >400 s^−1^ in all array sites.

In addition to loading site-selectively, it is also desirable to deterministically load a single ion into a given site. Therefore, inadvertent loading into occupied array sites must be minimized. This process can be due to the finite size of the PI beams (1/*e*^2^ radius ∼60 μm) extending into adjacent array sites or the presence of the weak 461 nm 2D-MOT push laser beam in the path of the 405 nm PI beam. We measured the incorrect-site loading probability of a particular ion trap site by looking for evidence of loading while attempting to load each adjacent site. The loading attempts consisted of 2 ms of PI and were repeated more than 50,000 times for each site. This probability was found to be ∼2 × 10^−4^ when loading the adjacent site along the 461 nm laser beam axis (lower right in Fig. 1c) and 7 × 10^−4^ when loading the adjacent site along the 405 nm laser axis (upper left in Fig. 1c). These inadvertent loading probabilities are expected to be strongly suppressed for sites farther from the loading site due to the orthogonal orientation of the PI beams. Under the assumption that inadvertent loading leads directly to qubit error, these probabilities are already sufficiently low for use with surface code error correction protocols^15^.

### Trapped-ion coherence measurements

To confirm our ability to maintain coherence of ions in all sites under the conditions necessary for rapid loading, we performed a series of Ramsey experiments on a single trapped ion in the presence and absence of neutral atom flux as well as each of the PI laser beams. Tests of the PI beams are necessary because although only a single site is simultaneously illuminated by both beams during loading, entire rows and columns of the trap array are subject to the beams individually. We also performed similar Ramsey experiments while loading adjacent trap sites. Each trial began with 1 ms of Doppler cooling, followed by resolved sideband cooling to the motional ground state of a 2.4 MHz radial trap mode. We then drove a 6.5-μs-long *π*/2 pulse on the narrow 5*S*_1/2_→4*D*_5/2_ transition at 674 nm. After a variable delay, we drove a second, phase-coherent *π*/2 pulse and measured the state of the ion. The Ramsey fringe contrast was determined by scanning the relative phase of the two *π*/2 pulses.

The coherence time in the absence of atomic flux and PI lasers was measured to be 480 μs. We verified that the coherence time was not limited by magnetic-field fluctuations by measuring the coherence time using two transitions with different magnetic-field sensitivities. The coherence times were found to be the same for both transitions, suggesting that such fluctuations did not limit our measurements. If the measured coherence decay is attributed solely to frequency fluctuations in the 674 nm laser driving the Ramsey pulses, we extract a laser linewidth of 1 kHz (ref. 16), which is consistent with direct spectroscopy of the *S*→*D* transition. With the inclusion of appropriately timed *π*-pulse spin echoes to counteract slow fluctuations, we are able to extend the 1/*e* coherence time beyond 3 ms. This technique is compatible with quantum information processing algorithms^17^ and allows us to measure decoherence on time scales comparable to the ion-loading time.

**Figure 3:**
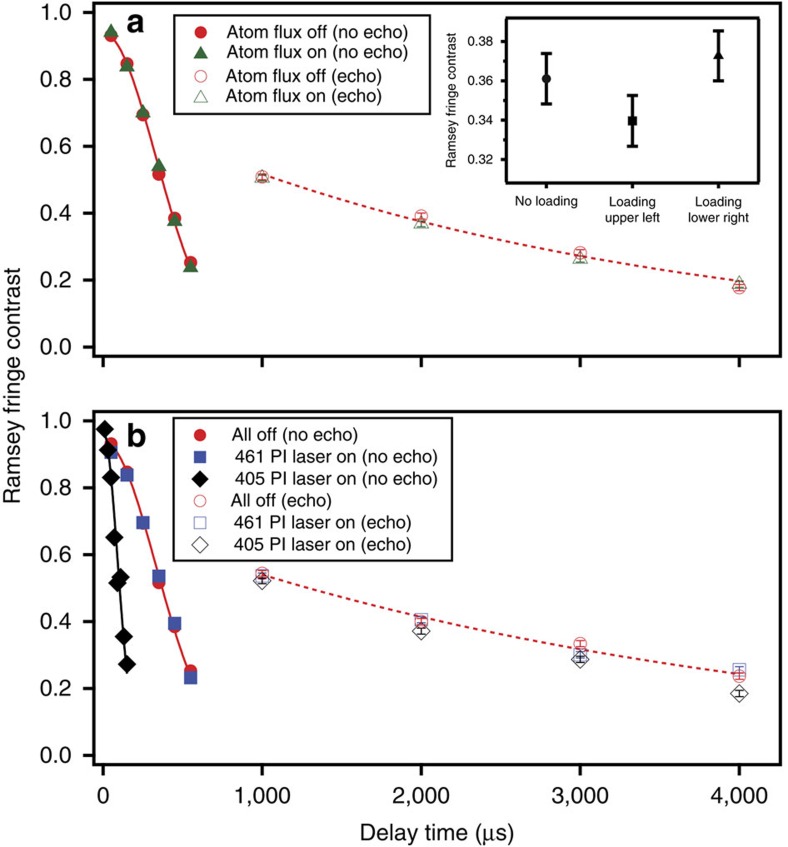
Coherence decay measurements performed under the conditions necessary to load site-selectively. (**a**) Measurements of the coherence in the presence and absence of neutral atom flux. (**b**) Coherence in the presence and absence of the 461 and 405 nm PI laser beams. For the longer delay times (open markers), spin-echo *π*-pulses were applied at *T*_0_, 3*T*_0_, 5*T*_0_ and 7*T*_0_, where *T*_0_=500 μs. Solid coloured lines are gaussian fits to the data markers of the same colour without using spin-echo pulses. Dashed lines are exponential fits to the measured data using spin-echo pulses in the absence of neutral atom flux and PI lasers. Error bars for all data points, which are comparable to the point size except in the inset, reflect s.e.m. from the Ramsey fringe contrast fit with quantum projection noise propagated throughout the fitting procedure. Each phase point trial was repeated 1,000 times for the primary figures and 500 times for the inset.

Figure 3a shows that the continuous flux of neutral Sr atoms had no measurable effect on the trapped-ion coherence at our current sensitivity for Ramsey delay times up to 4 ms. Collisions between atoms and the trapped ion are predicted to occur at a rate given by the product of the atomic flux *N*_Sr_ and the ion-atom collision cross-section *σ*. We have estimated the atomic flux by observing fluorescence from the atomic beam at the ion trap chip location in the cryogenic trapping chamber as *N*_Sr_≈10^8^ cm^−2^ s^−1^. The relevant Langevin cross-section can be calculated using the polarizability of Sr and the approximate atom velocity *v*≈70 m s^−1^ to yield *σ*≈3 × 10^−13^ cm^2^(refs 18, 19). Hence, we predict a collision rate and worst-case qubit error rate per ion of ∼3 × 10^−5^ s^−1^. Given the measured loading rates and trap lifetimes, such a collision probability would allow us to maintain arrays of ∼10^7^ ions in the presence of continuous atomic flux, conservatively assuming that each ion-atom collision results in ion loss, and also assuming that these collisions limit the maintainable array size.

As seen in Fig. 3b, the relatively weak intensity 461 nm PI beam had no effect on the trapped-ion coherence, as expected given its large detuning from all ^88^Sr^+^ transitions. When the ion was exposed to the 405 nm PI laser, however, a large reduction in Ramsey fringe contrast was observed. The AC Stark effect due to this much more intense beam (*I*_peak_=230 W cm^−2^) significantly shifts the 5*S*_1/2_ and 5*P*_3/2_ levels of the ion, whose transition is located near 408 nm. This level shift caused a 60 kHz detuning of the narrow 5*S*_1/2_→4*D*_5/2_ transition that required adjusting the 674 nm laser frequency to drive high-fidelity Ramsey pulses. However, even when the 405 nm laser was only on during the Ramsey delays and off during the pulses, the gaussian fit coherence time without spin-echo pulses was reduced to 130 μs from 480 μs.

We attribute the measured dephasing to low-frequency intensity noise, likely due to fluctuations in the 405 nm beam pointing. For an assumed gaussian distribution of pointing errors, the measured coherence time in the presence of the 405 nm laser corresponds to angular beam deviations at the final focusing lens of ∼90 microradians, which are consistent with measurements made using a quadrant photodiode. With the inclusion of spin-echo pulses to mitigate low-frequency fluctuations, the contrast in the presence of the 405 nm laser improved dramatically. Only a slight degradation of coherence relative to the measurements without the 405 nm laser was observed for delays up to 4 ms.

To measure trapped-ion coherence while attempting to load an adjacent trap site, the PI lasers were on for the duration of a 2 ms Ramsey experiment, which included two spin-echo *π*-pulses. Under these conditions, the loading probability is >50%, and the measurements were repeated 500 times per phase point. In these experiments, we observed different behaviour when loading the two sites adjacent to the upper-right site in Fig. 1c. As seen in the inset of Fig. 3a, the coherence was unaffected when loading the adjacent site along the 461 nm PI laser axis (lower right in Fig. 1c) for 2 ms. Given that neither the 461 nm laser nor atomic flux separately reduced the contrast, this result is consistent with the expectation that the 500 μm separation between array sites is large enough that the ion–ion Coulomb interaction is too small to perturb the nearby trapped ion. When loading the site along the 405 nm PI laser axis (upper left in Fig. 1c), we measured coherence consistent with what was observed when the 405 nm laser was applied in the absence of atomic flux.

## Discussion

In future work, we intend to scale the arrays to accommodate more ions. We estimate the array size that can be reloaded using this scheme by considering the Rayleigh length over which the PI beams remain sufficiently focused across the surface of the trap array. For an array pitch of 500 μm and beam waists of 60 μm for both PI lasers, site-selective loading of an array containing more than 10,000 ions may be possible. We also plan to further reduce any decoherence due to the 405 nm laser by stabilizing its intensity or by implementing integrated photonic grating couplers^20^ to address only a single array site during loading. In addition, grating couplers will reduce the already small probability of inadvertently loading into occupied sites and will permit parallel loading of array sites without additional atomic flux; this approach may also allow loading of much larger arrays by eliminating the limitations set by the finite Rayleigh length of the PI beams.

In conclusion, we have demonstrated a surface-electrode ion trap loading scheme designed to site-selectively address individual traps within an array. By photo-ionizing neutral atoms from a continuous, pre-cooled atomic beam, we measured average loading rates >400 s^−1^ for each site of a 2 × 2 array while maintaining trap lifetimes of many hours. Importantly, no reduction in trapped-ion Ramsey fringe contrast was observed in the presence of neutral atomic flux from our 2D-MOT system. Hence, the oven can operate continuously, affording the fastest random-access loading rates. Exposure to the intense 405 nm PI laser beam used in site-selective loading was seen to dephase the ion qubit, but this effect was almost entirely eliminated with the use of straightforward spin-echo techniques. Measurements while attempting to load adjacent trap sites showed no additional trapped-ion decoherence beyond what was caused by the 405 nm laser. Hence, with the inclusion of spin-echo pulses, we have demonstrated site-selective array reloading with minimal dephasing of a nearby qubit in a way that satisfies the major criteria for scalable operation of a large trapped-ion quantum information processor.

## Methods

### Trap chip fabrication

**Figure 4:**
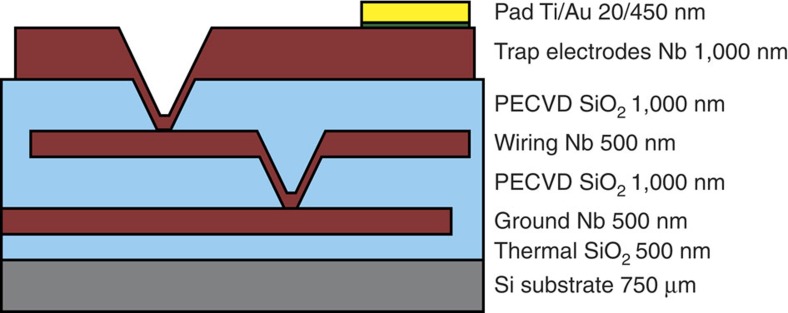
Multilayer stack of trap chip (not drawn to scale). Surface electrodes, wiring layer and ground plane are made of sputtered Nb. Insulating layers are formed by plasma-enhanced chemical vapour deposited silicon dioxide (SiO_2_).

The 1 × 1 cm trap chip was fabricated on a 200 mm silicon wafer substrate using a superconducting-multilayer process as shown in Fig. 4 and performing standard optical lithography and etching techniques. The trap electrode layer and the wiring layer below it were made from sputtered niobium (Nb) and were insulated by interspersed layers of silicon dioxide deposited using plasma-enhanced chemical vapour deposition. An additional Nb ground plane was deposited between the wiring layer and the silicon substrate to prevent optically generated charge carriers within the silicon from affecting the trap impedance^21^. Electrical connections to the trap electrodes were made using interlayer Nb vias contacting the wiring layer. All Nb layers were defined using SF_6_ plasma etching. The wiring layer was routed to gold pads at the edge of the chip that were wire-bonded to a ceramic pin grid array chip carrier using gold bond wire. The ceramic pin grid array chip carrier was mounted using a zero-insertion force socket to a Rogers electronic filter board that was attached to the cold head of the cryogenic system. In this configuration, the trap reached a steady-state temperature of ∼8 K at the highest RF amplitude used here.

### Two-dimensional magneto-optical trap

Ions are loaded from a cold neutral atomic beam produced remotely in the two-dimensional magneto-optical trap (2D-MOT) chamber and accelerated to the trap array. The MOT is produced using two retro-reflected laser beams detuned by ∼60 MHz from the ^88^Sr ^1^*S*_0_→^1^*P*_1_ transition (natural linewidth: 32 MHz). The MOT beams are ∼2.5 cm in diameter with a combined power of ∼50 mW, and they intersect one another at the centre of the MOT chamber. Stacks of permanent magnets fastened to the outside of the chamber produce a magnetic-field gradient with a maximum amplitude of ∼50 G cm^−1^. An oven attached to the MOT chamber heats a sample of Sr metal to ∼400 °C; Sr atomic vapour is emitted from the oven's nozzle which consists of ∼25 high-aspect-ratio capillaries to reduce the transverse velocity spread of the emitted atoms^22^,^23^. Atoms are cooled in the MOT cloud to energies equivalent to a few milliKelvin in the plane containing the four MOT beams. The atoms are untrapped in the direction perpendicular to this plane—they are accelerated in this direction, through a long, narrow constriction (inner diameter: 2 mm; aspect ratio: ∼12) between the MOT chamber and the main chamber, and toward the trap chip, by means of a ‘push' laser beam tuned near resonance with the ^1^*S*_0_→^1^*P*_1_ transition. The constriction allows for considerably lower pressures in the cryo-pumped main chamber when compared with the MOT chamber.

### Data availability

All relevant data presented in this paper are available from the corresponding author on request.

## Additional information

**How to cite this article:** Bruzewicz, C. D. *et al*. Scalable loading of a two-dimensional trapped-ion array. *Nat. Commun.* **7,** 13005 doi: 10.1038/ncomms13005 (2016).

## Figures and Tables

**Table 1:** Average loading times and rates for each array site.

**Array site**	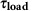 **(ms)**	**Loading rate (s**^−1^**)**
Upper right	1.80(6)	560(20)
Upper left	1.95(5)	510(10)
Lower right	2.37(5)	420(10)
Lower left	2.47(6)	410(10)

Values in parentheses reflect uncertainties from the model fit.
